# A brief research report on the efficacy of Griffonia and Lactium supplementation in a clinical preschool sample with anxiety disorders and emotional dysregulation

**DOI:** 10.3389/frcha.2026.1905412

**Published:** 2026-10-07

**Authors:** Paola Linguanti, Irene Troiano, Marika Ricci, Greta Tolomei, Roberta Igliozzi, Antonio Narzisi, Gabriele Masi, Gianluca Sesso, Valentina Viglione

**Affiliations:** 1 IRCCS Stella Maris Foundation, Pisa, Italy; 2 IMT School for Advanced Studies, Lucca, Italy

**Keywords:** anxiety treatment, emotional dysregulation, Griffonia, Lactium, nutraceuticals, preschoolers

## Abstract

Anxiety in pre-schoolers predicts the persistence of anxiety during school age and mid-adolescence and may also interfere with later social and academic functioning. Currently, while no pharmacological treatments are specifically approved for anxiety disorders in the preschool population, novel nutraceutical compounds with potential anxiolytic effects have received increasing research attention. We conducted an exploratory single-center, longitudinal, interventional study aimed at evaluating the efficacy of a 3-month supplementation with a dietary supplement containing Griffonia and Lactium in 22 preschool children (3–6.11 years; mean age 4.6 ± 0.8 years) diagnosed with an anxiety disorder, either as a primary condition or comorbid with other internalizing and externalizing disorders, particularly emotional dysregulation. A small subpopulation of the sample continued supplementation for a total of 6 months, and efficacy outcomes were compared with those observed in participants who received the supplement for only 3 months. Additionally, maintenance of the effects was assessed at a follow-up evaluation conducted 3 months after discontinuation of the dietary supplement. Efficacy of the dietary supplementation at predefined time points was evaluated using both a parent-reported questionnaire (Preschool Anxiety Scale—Parent Report, PAS) and clinician-rated scales derived from available data, including clinical observation (Children's Global Assessment Scale, CGAS and Clinical Global Impression, CGI). The results of the present study suggest that 3 months of supplementation with a Griffonia- and Lactium-based dietary supplement, in addition to demonstrating a favorable safety profile, may be associated with improvements in anxiety symptoms and overall functioning. These effects appear to be maintained up to three months after treatment discontinuation. Moreover, a longer duration of dietary supplement administration, lasting six months, may appear to be associated with more sustained clinical improvement over time. Although this study provides a relevant contribution to an underexplored area of research in the preschool population, the findings should be considered exploratory and further validation through randomized controlled trials is required.

## 1 Introduction

Anxiety disorders are among the most common mental health problems in children and adolescents. Nearly 7% of youths worldwide have an anxiety disorder and at least one-third of them meet the criteria for two or more subtypes of anxiety disorders (1, 2). Moreover, these disorders commonly occur with other psychiatric conditions, such as neurodevelopmental disorders (ADHD and ASD in particular) and mood disorders, often associated with heightened distress and functional impairment (3–5). Increasing evidence indicates that many anxiety disorders have their onset in early childhood. The preschool (3–6 years) anxiety disorders may persist or recur and also predict the stability of anxiety in school-age/mid-adolescence (6–8). Therefore, if untreated, these disorders tend to follow a chronic course and increase the risk of developing other psychiatric conditions (9–11). Less than 15% of young children with an impairing anxiety disorder receive a mental health evaluation or treatment. The ability to quickly and reliably detect and manage anxiety disorders, while the child's brain is still developing, could directly improve the child's developmental trajectory (12, 13). For these reasons, early diagnosis is essential to ensure the timely implementation of targeted therapeutic interventions. Beyond categorical diagnostic assessment, as the DSM5-TR model (14), especially in pediatric populations, it remains important to identify dimensional manifestations common across various anxiety disorders, which often constitute the primary reason for seeking specialized consultation. These can include frequent and intense episodes of emotional and behavioral dysregulation, such as screaming, crying, and both self- and other-directed aggression (15–19). Moreover, many preschool anxiety-related manifestations may overlap with typical developmental behaviors, with the risk of diagnostic delays. Nevertheless, through a specific psychiatric assessment, it is possible to distinguish clinically significant symptoms from “extreme” but developmentally appropriate behaviors (9, 12, 20). Evidence-based assessment incorporates a variety of methods (clinical interviews, rating scales, observations) and includes information from multiple sources who know the child well, such as caregivers and teachers as well as the youth himself or herself, for older preschoolers (4, 21).

The etiology of these disorders involves a complex interaction between biological and environmental risk factors. Among neurobiological factors, anxiety disorders involve both brain circuits associated with the amygdala (the “fear circuit”) and the cortico-striato-thalamo-cortical (CSTC) pathways (the “worry circuit”). Both systems are modulated by various neurotransmitters, with particular involvement of the serotonergic (5-HT) and GABAergic (GABA) systems (22). CBT, tailored to developmental level, with the involvement of parents, is considered to be the first-line treatment in very young children. Currently, there are no pharmacological treatments approved with a specific indication for anxiety disorders in the preschool population (4, 9). Given the limited pharmacologic data in preschool children with psychiatric disorders, novel nutraceutical compounds with potential preventive and/or therapeutic effects on psychiatric and neurodevelopmental disorders have attracted increasing research interest in recent years. Several reviews and meta-analyses have investigated the use of nutraceuticals, phytoceuticals, and probiotics (including omega-3 fatty acids, vitamin D, vitamins B1, B6, and B12, micronutrients) in both pediatric and adult populations, across various psychiatric disorders, such as schizophrenia, autism spectrum disorder, attention-deficit/hyperactivity disorder, mood disorders, personality disorders, and anxiety disorders. Although the findings are promising in terms of clinical efficacy and tolerability, the evidence remains heterogeneous, partly because of substantial differences in study designs, sample sizes, and dosages administered. The use of nutraceuticals in psychiatric and neurodevelopmental disorders therefore remains an active area of research. Even if recent literature has highlighted a growing interest in the pediatric population, further studies with greater methodological standardization and larger sample sizes are needed to establish more robust clinical recommendations regarding their use, particularly in preschool populations (23–25). The possibility of combining targeted rehabilitative interventions in preschool children with anxiety disorders and supplementation aimed at modulating the involved neurotransmitter systems may provide significant support in improving the child's overall functioning. Among various nutraceutical products that may have beneficial effects on anxiety symptoms, increasing attention has recently been given to casein derivatives and Griffonia seeds, which are rich in tryptophan (Trp) (26), an essential amino acid that serves as a metabolic precursor of serotonin, melatonin, and niacin (vitamin B3). 5-Hydroxytryptophan (5-HTP), a metabolite of tryptophan, is a direct precursor of both serotonin and melatonin and has already been used in the treatment of sleep disorders and anxiety-depressive syndromes. Furthermore, the efficacy of lactium (an industrial hydrolysate of *α*s1-casein containing the bioactive peptide *α*-casozepine) has been reported in adult women with stress-related symptoms (27), with anxiolytic and pro-hypnotic effects (28).

We conducted an exploratory single-center, longitudinal, interventional study in order to evaluate the efficacy of a 3-month supplementation with a dietary supplement containing Griffonia and Lactium in 22 preschool children (3–6.11 years; mean age 4.6 ± 0.8 years) with an anxiety disorder, either as a standalone condition or in association with other internalizing and externalizing disorders, particularly emotional dysregulation. Additionally, we aimed to assess the maintenance of symptom improvement 3 months after discontinuation of the dietary supplement.

## 2 Materials and methods

### 2.1 Study design

The study was conducted in accordance with the initial protocol (Version 1, 9 May 2022) and its reviews approved by the Ethics Committee on 21 February 2024 (Version 2, 13 November 2023). Preschool children with a diagnosis of an anxiety disorder, either as a primary condition or comorbid with other internalizing and externalizing disorders, were eligible for this study. In the final protocol was emphasized emotional dysregulation, based on established clinical evidence and preliminary study data suggesting that anxiety symptoms in preschool-aged children often manifest primarily as emotional and behavioral dysregulation rather than developmentally typical anxiety presentations. Participants receiving concomitant psychological and/or educational interventions were eligible for inclusion, provided that the frequency of such interventions remained unchanged for at least three months following study enrollment. Exclusion criteria included an Intelligence Quotient (IQ) below 70, lactose intolerance, and concomitant pharmacological treatment.

Participants were withdrawn from the study in the event of: (1) clinical worsening requiring the initiation of disorder-specific pharmacological treatment; (2) withdrawal of parental consent or (3) the occurrence of adverse events temporally associated with the intake of the dietary supplement. To date, the only reported risk associated with excessive intake of the investigational supplement is a mild laxative effect.

After obtaining informed consent from caregivers, the study comprised three sequential phases: (1) an initial diagnostic assessment, (2) a clinical monitoring phase, and (3) a long-term follow-up phase. During the first phase, participants underwent a comprehensive psychiatric assessment using a diagnostic protocol routinely employed in clinical practice for preschool-aged children. Children meeting all inclusion criteria and for whom initiation of treatment with the study dietary supplement was clinically indicated proceeded to the second and third phases.

In the first protocol version, the dietary supplement was administered for 3 months at a daily dosage of 75 mg Griffonia (providing 15 mg 5-hydroxytryptophan) and 150 mg hydrolyzed milk protein. In the second version of the protocol, the same daily dosage was administered for 6 months. For the efficacy analysis, only data from the 3-month endpoint were considered for both groups. The long-term follow-up phase included an assessment conducted three months after discontinuation of dietary supplementation in both versions of the protocol.

The primary endpoint of the study was to evaluate the efficacy of the dietary supplement in reducing anxiety symptoms after 3 months of treatment. The secondary objective was to assess the maintenance of improvement in the outcome measures at 3 months following discontinuation of the dietary supplementation.

In a separate observational analysis, not included in the study protocol, we compared the developmental trajectory of children who received the dietary supplement with the retrospectively reconstructed trajectory of a comparison group of 15 preschool children (3.0–5.11 years; mean age 4.4 ± 0.8 years). This sample was recruited in a naturalistic clinical setting between 2021 and 2024. All had anxiety symptoms and had not received the supplement under investigation.

### 2.2 Diagnostic procedure and clinical measures

- Interview for the psychopathological evaluation in childhood (IVPI 3-6) (29): a semi-structured interview administered to parents, aimed at assessing psychopathology in children aged 3 to 6 years, according to DSM-5 criteria.
- Preschool Anxiety Scale—Parent Report (PAS-PR) (30): a parent-report questionnaire to assess various dimensions of anxiety in young children. It includes five subscales: Generalized Anxiety Disorder (GAD), Social Anxiety (SA), Obsessive-Compulsive Disorder (OCD), Fears of Physical Injury (PiF), and Separation Anxiety Disorder (SAD). The scale contains 28 items, each rated from 0 (“Not at all true”) to 4 (“Very often true”). The Italian version of the questionnaire was translated into Italian by our research team and approved by the author.
- Clinical Global Impression (CGI) (31, 32): an overall clinician-determined summary measure that takes into account all available information, including knowledge of the patient's history, symptoms and the impact of symptoms on the patient's functioning. The CGI comprises two companion one-item measures evaluating the following: (a) severity of psychopathology from 1 to 7 (1 = normal, not at all ill; 7 = among the most extremely ill patients) and (b) change since treatment initiation on a similar seven-point scale (1 = very much improved since treatment initiation; 7 = very much worse since treatment initiation).
- Children's Global Assessment Scale (C-GAS) (33): provides a global measure of the severity of psychiatric illness and level of functional impairment in social, familial and school domains. Scores range from a minimum of 1 (persistent danger of causing serious harm to oneself or others) to a maximum of 100 (superior functioning across a wide range of activities; no symptoms).

### 2.3 Statistical analysis

All statistical analyses were performed using RStudio software (RStudio, Vienna, Austria). To evaluate longitudinal changes over time irrespective of group allocation, linear mixed models (LMMs) were first fitted for each continuous outcome (e.g., PAS subscales, CGAS scores) using the lme4 package. Timepoint (T0 versus T1 versus T2) was included as a fixed effect, with a random intercept for subject identity to account for within-subject correlation. Predefined contrasts between timepoints were extracted. To subsequently investigate potential differential effects between groups, pairwise LMMs were conducted including the interaction between Timepoint (T0 versus T1 versus T2) and Group (3 Months versus 6 Months). These models allowed estimation of group-specific trajectories over time. Additional models were fitted to control for potential confounding variables, including age, gender, and neurodevelopmental comorbidities (ADHD and ASD diagnoses), as well as interaction terms between Timepoint and each covariate, which were included to explore their moderating effects on outcome trajectories. For all mixed-effects models, parameters were estimated using maximum likelihood, and statistical inference was based on Wald t tests with Satterthwaite approximation for degrees of freedom (lmerTest package). Given the large number of outcomes and comparisons, *p* values were adjusted for multiple testing using the false discovery rate (FDR) correction (Benjamini-Hochberg procedure). Since CGI-S scores were not available across each timepoint, non-parametric between-group comparisons of CGI-S at T0 and of CGI-I at follow-ups (T1 and T2) were performed using Wilcoxon rank-sum test. Finally, exploratory analyses further examined differences between subjects undergoing dietary supplement as a whole versus subjects who had not received the supplement under investigation. For these models that included Timepoint × Group interaction term, Analysis of Variance (ANOVA) was used to derive F statistics and associated *p* values for interaction effects, with FDR correction applied. All statistical tests were two-tailed, and statistical significance was set at *p* < 0.05 after FDR correction for multiple comparisons.

## 3 Results

Between May 2022 and January 2025, 25 children who met the required inclusion criteria and whose parents provided written informed consent were enrolled in the study. Three participants were not included in the statistical analysis due to very early dropout: one participant because of a worsening of symptoms requiring specific pharmacological treatment, and two participants due to parental withdrawal of consent. All children presented with anxiety disorder. Comorbidities were predominantly neurodevelopmental disorders: ADHD (86%), ASD (45%), and language disorder (23%). In addition, 68% of the children exhibited symptomatology consistent with emotional dysregulation, and 9% had a diagnosis of oppositional defiant disorder. No other psychiatric comorbidities were identified in the sample. Of the 22 children who participated in the study, 8 were enrolled with the first version of the protocol, which involved 3 months of dietary supplementation, whereas 14 were enrolled with the second version, whose main modification was the extension of supplementation to 6 months. In the second version of the protocol, follow-up assessments were maintained at 3 months after the initiation of dietary supplementation and at 3 months following its discontinuation, according to the same timeline as the first version of the study.

Analyses conducted on the sample of 22 children (8 females and 14 males) to evaluate longitudinal changes in outcome measures at the defined timepoints showed that, during the 3-month administration of the dietary supplement, there was a statistically significant reduction in the “PAS-Total Score” (*β* = −5.0455; t = −3.0138; *p*-value = 0.03), in the PAS subscales “Social Anxiety” (*β* = −6.2273; t = −3.9566; *p*-value = 0.004) and in the “Fears of Physical Injury” (*β* = −4.9091; t = −2.9674; *p*-value = 0.03). A statistically significant reduction was maintained at the assessment performed three months after supplement discontinuation for “PAS-Total Score” (*β* = −6.2299; t = −3.2194; *p*-value = 0.02) and “Social Anxiety” subscale score (*β* = −6.7165; t = −3.6884; *p*-value = 0.009). These findings derive from analyses conducted on a smaller sample (*n* = 16), as six participants dropped out prior to the post-discontinuation follow-up assessment (Figure 1).

**Figure 1:**
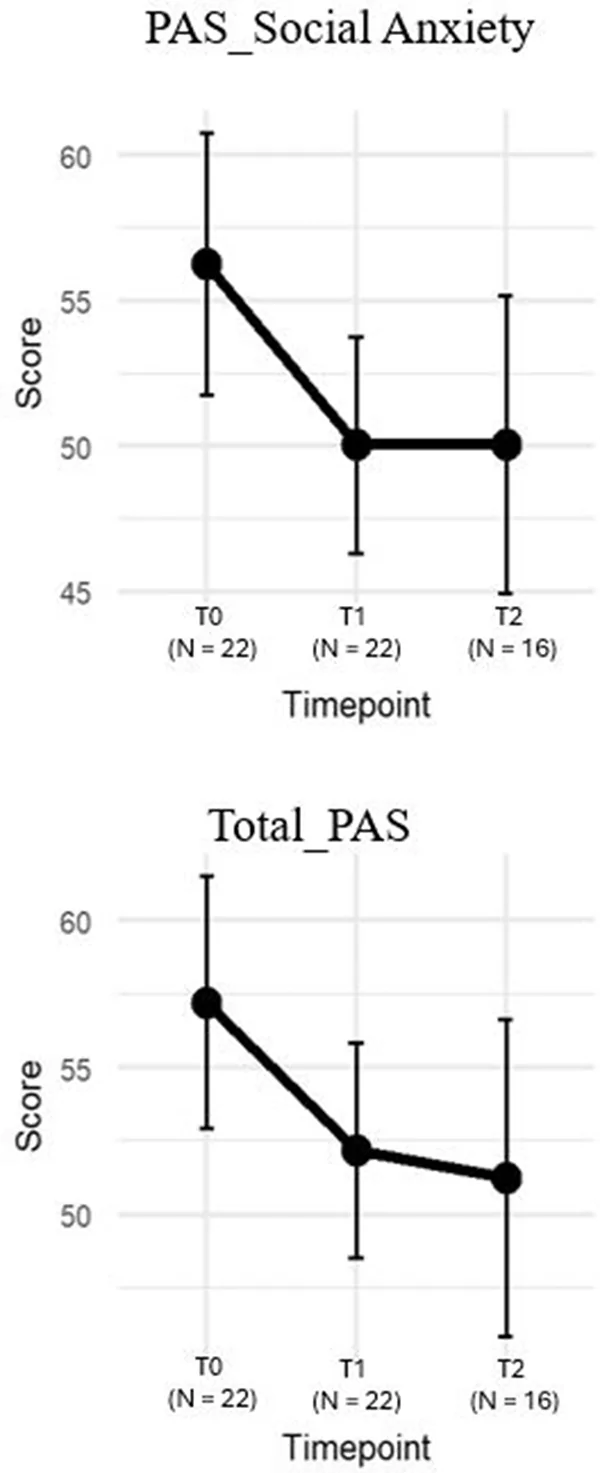
Longitudinal improvement in the PAS total score and in the PAS “social anxiety” subscale following 3-month administration of the dietary supplement (T1) maintained at the 3-month post-discontinuation assessment (T2).

Based on the CGAS (Children's Global Assessment Scale), a statistically significant increase in clinician-rated scores was observed after 3 months of supplementation (*β* = 7.7273; t = 6.0476; *p*-value < 0.0001). This increase was maintained at the follow-up assessment conducted 3 months after discontinuation of the supplement (*β* = 6.9754; t = 4.8375; *p*-value = 0.0005).

The interaction between timepoints and other covariates of interest (e.g., gender and diagnoses of ADHD and ASD—those most represented in our sample) was subsequently analyzed, yielding the following results. With regard to gender differences, at the follow-up assessment conducted three months after discontinuation of dietary supplementation, females showed a significantly greater improvement in CGAS scores compared with males (*β* = −8.2893; t = −3,4603; *p*-value = 0.04).

Analyses conducted to examine potential differential effects between groups (preschoolers who received the supplement for three months vs. those who received the supplement for six months) did not yield statistically significant results. However, the mean CGI-I scores of children who received the supplement for 3 months were significantly higher than those of children who received the supplement for 6 months (M = 3.38; SD = 1.2; W = 54.5; *p*-value = 0.02) after three months of discontinuation of the dietary supplement (Figure 2).

**Figure 2:**
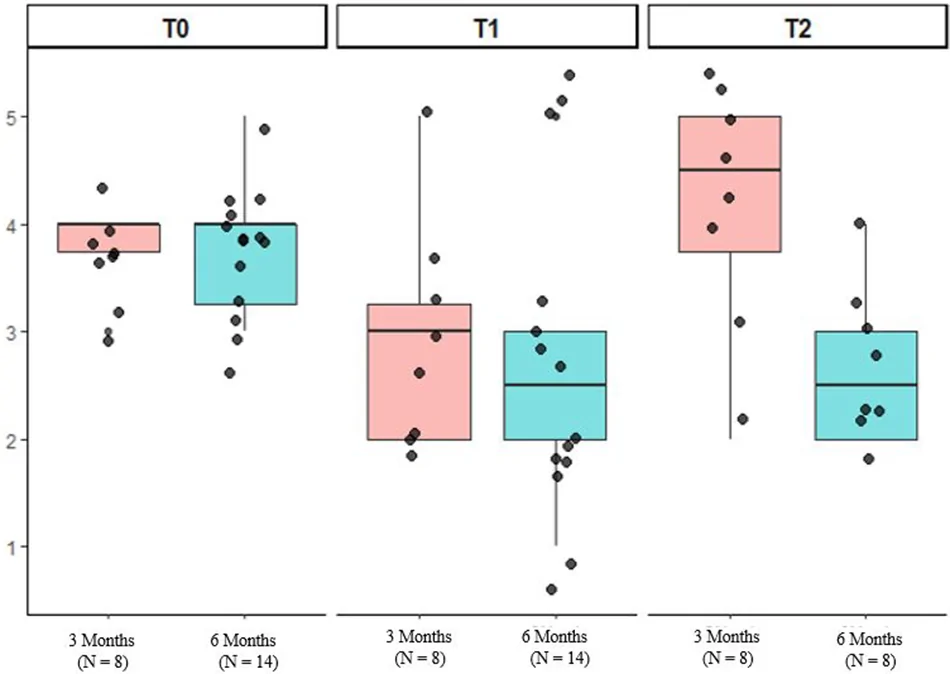
Comparison of CGI-S at baseline (T0) and CGI-I at follow-ups (T1 and T2) between groups (3-month vs. 6-month supplementation).

No statistically significant differences were found according to the administration of a rehabilitative intervention (psychomotor or psychotherapeutic).

Finally, from the comparison of clinical trajectories between the group of preschool children who received the dietary supplement and a sample of 15 preschool children with anxiety symptoms who had not received the supplement under investigation, it emerged that, at the end of the observation period, children who had taken the dietary supplement obtained higher scores on the CGAS compared with those who had not received the supplement (F = 15.15; *p*-value = 0.01).

No adverse events were observed in any patients during the observation period; nevertheless, six patients dropped out of the study: four due to withdrawal of parental consent, one due to worsening clinical symptoms, and one due to difficulties in supplement administration to taste acceptability.

## 4 Discussion

Preschool anxiety has been shown to persist into adolescence and adulthood and to be associated with an elevated risk of developing other mental health problems (3, 10, 19). For these reasons, early diagnosis is crucial to ensure the timely implementation of targeted therapeutic interventions. Currently, no pharmacological treatments are specifically approved for anxiety disorders in the preschool population. In recent years, novel nutraceutical compounds with potential anxiolytic effects have attracted increasing attention (4, 27, 28), although the scientific evidence in this field remains limited and is largely derived from studies conducted in adults. Moreover, studies included in recent reviews and meta-analyses concerning the pediatric age group more often involve populations with neurodevelopmental disorders (ADHD and ASD in particular), in which the efficacy of fatty acids and/or probiotics is more frequently assessed (23, 34, 35). Nevertheless, a limited number of studies have reported improvements in anxiety symptoms in pediatric populations with other mental disorders (e.g., ADHD and tic disorders) following treatment with nutraceuticals, either as monotherapy or in combination (e.g., magnesium and vitamin D, L-theanine, etc) (36, 37).

To contribute to this still underexplored area of scientific research on preschool populations, we conducted a longitudinal, exploratory study in a small sample of children aged 3 years to 6 years and 11 months with an anxiety disorder, either as a standalone condition or in association with other internalizing and externalizing disorders, particularly emotional dysregulation. The primary endpoint of the study was to evaluate the efficacy of a 3-month supplementation with a dietary supplement containing Griffonia and Lactium.

Before describing the study findings, we highlight some limitations that should be considered when interpreting the results. The small sample size and the number of dropouts occurring during the study period must be acknowledged. Furthermore, the high rate of psychiatric comorbidity — expected in a tertiary care hospital setting — predominantly involving neurodevelopmental disorders, may have contributed to partial symptom overlap, thereby limiting the generalizability of the findings. Nevertheless, these aspects (small simple size, high comorbidity rate) should also be considered in relation to the characteristics of the specific clinical sample. Indeed, during the preschool age, complex symptom presentations are very common, with high levels of emotional and behavioural dysregulation, hyperactivity and impulsivity. Therefore, the clinical heterogeneity of the sample reflects real-world clinical practice, as opposed to highly selected samples, which are less representative of routine clinical settings. Finally, the relatively short-term follow-up of the study, the different assessment time points across the two protocols, and the absence of a control group represent methodological constraints. As mentioned above, this was an exploratory study with an open-label, single-arm design without placebo control, and the results should be interpreted with caution.

Despite the limitations discussed above, the study has notable strengths, including the use of outcome variables derived from both clinical observations and parent reports. Assessment in the preschool years should, in fact, combine direct clinical observation with information from adults who interact with the child in other settings and spend a substantial amount of time with them (4); children, may display different aspects of their behavior and personality depending on the relational context.

Taking into account the limitations of the study described above, the findings after 3 months of dietary supplementation showed a significant improvement not only in global anxiety levels but also in specific symptom domains, particularly relevant in preschool-aged children, such as social anxiety and specific phobias. Indeed, a significant reduction in the scores of the “social anxiety” and “fears of physical injury” subscales was observed, as well as in the total score of the PAS questionnaire completed by parents after 3 months of dietary supplementation. A significant improvement in global functioning, as assessed by the clinician using the CGAS scale, was also observed after 3 months of dietary supplementation.

The improvements reported by parents in global anxiety levels and social anxiety, as well as the improvement in global functioning assessed by the clinician, appeared to persist at follow-up, 3 months after discontinuation of the supplement.

Consistent with the existing literature on anxiety disorders in preschool-aged children, this study found no statistically significant gender differences in the expression of anxiety symptoms over the study period (10, 30). Nevertheless, females maintained improvements in overall functioning to a significantly greater extent than males throughout the observation period.

With respect to the duration of supplementation, children who received the dietary supplement for 6 months showed significantly lower scores on the clinician-rated CGI-I at the 3-month post-discontinuation follow-up. This finding may reflect a more persistent improvement in children treated for 6 months compared with those who received supplementation for 3 months.

Finally, exploratory analyses were conducted to investigate potential differences between subjects who received dietary supplementation and preschoolers with anxiety disorders who did not receive the dietary supplement under investigation. The comparison group consist of 15 preschool children (3.0–5.11 years) with anxiety symptoms and had not received the supplement under investigation, recruited in a naturalistic clinical setting. For the comparative analysis, data obtained from the review of medical records were used. The results of these analyses should be considered purely exploratory, as they refer to two non-contemporaneous, non-randomized groups, whose data were collected using prospective and retrospective methods, respectively. Despite the aforementioned limitations, the results showed significantly better global functioning in the group of preschoolers who had received the dietary supplement, at the end of the respective observation periods.

Therefore, although it is not possible to establish a causal relationship between dietary supplement intake and improvement in anxiety symptoms and overall functioning, the present findings suggest that the supplement under investigation may have contributed to such improvement. These results are consistent with previous literature, including both preclinical and clinical studies, that has reported beneficial effects on anxiety and stress associated with the individual administration of Griffonia and Lactium (26–28). Furthermore, the observed improvements in our sample of preschool children, despite the relatively short observation period and the small sample size, may be particularly noteworthy in light of the developmental trajectory of early-onset psychiatric disorders, which has been shown to exhibit substantial homotypic and heterotypic continuity from preschool to school age, particularly in the domain of anxiety disorders, including social anxiety and specific phobias (10). The ability to intervene rapidly and effectively in anxiety disorders at an early developmental stage may indeed directly improve the child's developmental trajectory (12, 13). Finally, no statistically significant differences were observed on the variables analyzed at the timepoints of the study based on whether participants had undergone psychomotor or psychological rehabilitation treatment. We may therefore hypothesize that rehabilitative intervention and nutraceutical treatment may act independently in contributing to clinical improvement in preschool children with an anxiety disorder. However, when interpreting this finding, should take into consideration the absence of some relevant information, such as the frequency and the overall duration of the treatment provided.

In conclusion, the presence of anxiety symptoms in the preschool years—particularly when associated with neurodevelopmental disorders—may interfere with the social challenges typical of this developmental stage (e.g., the transition to kindergarten) and may manifest through symptoms linked to physical stimuli, plausibly reflecting also separation anxiety. Improvement in these symptoms can have a significant impact on the child's emotional well-being, self-esteem, and ability to engage in social opportunities. For this purpose, the possibility of combining the rehabilitative intervention with nutraceutical compounds with potential anxiolytic effects may provide significant support in improving the child's overall functioning. Although significant within a relatively unexplored area of research, these findings should be considered preliminary and exploratory. Further validation through randomized controlled trials with larger samples and longer follow-up periods is required to provide more robust findings and enhance the generalizability of the results.

## Data Availability

The datasets presented in this article are not readily available because the dataset contains sensitive/clinical data and is not publicly available due to privacy and ethical restrictions. De-identified data may be available from the corresponding author upon reasonable request, subject to ethical approval. Requests to access the datasets should be directed to valentina.viglione@fsm.unipi.it.
